# Integrated therapy for HIV and tuberculosis

**DOI:** 10.1186/s12981-016-0106-y

**Published:** 2016-05-12

**Authors:** Weerawat Manosuthi, Surasak Wiboonchutikul, Somnuek Sungkanuparph

**Affiliations:** Department of Disease Control, Ministry of Public Health, Bamrasnaradura Infectious Diseases Institute, Nonthaburi, Thailand; Division of Infectious Diseases, Department of Medicine, Faculty of Medicine Ramathibodi Hospital, Mahidol University, 270 Rama 6 Road, Bangkok, 10400 Thailand

**Keywords:** HIV, Tuberculosis, Treatment, Integrated therapy

## Abstract

Tuberculosis (TB) has been the most common opportunistic infection and cause of mortality among HIV-infected patients, especially in resource-limited countries. Clinical manifestations of TB vary and depend on the degree of immunodeficiency. Sputum microscopy and culture with drug-susceptibility testing are recommended as a standard method for diagnosing active TB. TB-related mortality in HIV-infected patients is high especially during the first few months of treatment. Integrated therapy of both HIV and TB is feasible and efficient to control the diseases and yield better survival. Randomized clinical trials have shown that early initiation of antiretroviral therapy (ART) improves survival of HIV-infected patients with TB. A delay in initiating ART is common among patients referred from TB to HIV separate clinics and this delay may be associated with increased mortality risk. Integration of care for both HIV and TB using a single facility and a single healthcare provider to deliver care for both diseases is a successful model. For TB treatment, HIV-infected patients should receive at least the same regimens and duration of TB treatment as HIV-uninfected patients. Currently, a 2-month initial intensive phase of isoniazid, rifampin, pyrazinamide, and ethambutol, followed by 4 months of continuation phase of isoniazid and rifampin is considered as the standard treatment of drug-susceptible TB. ART should be initiated in all HIV-infected patients with TB, irrespective of CD4 cell count. The optimal timing to initiate ART is within the first 8 weeks of starting antituberculous treatment and within the first 2 weeks for patients who have CD4 cell counts <50 cells/mm^3^. Non-nucleoside reverse transcriptase inhibitor (NNRTI)-based ART remains a first-line regimen for HIV-infected patients with TB in resource-limited settings. Although a standard dose of both efavirenz and nevirapine can be used, efavirenz is preferred because of more favorable treatment outcomes. In the settings where raltegravir is accessible, doubling the dose to 800 mg twice daily is recommended. Adverse reactions to either antituberculous or antiretroviral drugs, as well as immune reconstitution inflammatory syndrome, are common in patients receiving integrated therapy. Early recognition and appropriate management of these consequences can reinforce the successful integrated therapy in HIV-infected patients with TB.

## Background

Tuberculosis (TB) is one of the most common opportunistic infections among HIV-infected patients especially in resource-limited countries worldwide. In Africa and Asia, TB is also the most common cause of mortality in HIV-infected population [1]. HIV changes the clinical presentation of TB from a slowly progressing disease to one with a high mortality rate. A prospective multicenter cohort has clearly demonstrated that the risk of TB is associated with increasing immunodeficiency and TB occurrence significantly increases the risk of mortality [2]. Optimal treatment of TB with good patient compliance can lead to the successful treatment and reduction of mortality [3].

Antiretroviral therapy (ART) has proven to have a great impact on survival rates among HIV-infected patients with TB [2, 4, 5]. Simultaneous treatment of both TB and HIV is required for HIV-infected patients presenting with TB. In addition, patients in resource-limited settings with low CD4 cell counts, continue to present late to healthcare providers. New episodes of opportunistic infections may develop within the first few months after ART and TB presents most frequently in this situation [6, 7]. An integrated therapy of both HIV and TB based on the current evidence of studies from both diseases has shown to be feasible and efficient in controlling the diseases and yields better survival in various clinical settings. This article focuses on the integrated therapy for HIV-infected patients with active TB and details regarding diagnosis and treatment of TB, initiation of ART, management of drug–drug interaction, overlapping toxicities of antituberculous and antiretroviral drugs, as well as TB immune reconstitution inflammatory syndrome are discussed.

### Clinical manifestations of TB in HIV-infected patients

TB among HIV-infected patients is resulted from reactivation of past infection or new infection of *Mycobacterium tuberculosis* [8]. Clinical manifestations are varying from classic symptoms of prolonged fever, hemoptysis, productive cough, weight loss, or night sweat to minimal or nonspecific symptoms [9]. A previous study in Southeast Asia had reported that the presence of cough of any duration, fever of any duration, or night sweats lasting 3 or more weeks in the preceding 4 weeks was 93 % sensitive and 36 % specific for tuberculosis [10]. The clinical features of TB depend on the degree of immunodeficiency. The presentation of pulmonary TB in AIDS patients may be atypical and unusual [11]. TB patients with CD4 cell counts less than 200 cells/mm^3^ are likely to have hilar or mediastinal adenopathy on chest radiographs, but less likely to have cavitary lesion [12]. Miliary infiltrate is commonly found on chest radiographs among AIDS patients [13]. Normal radiographs can be found in 14–22 % of HIV-associated TB [14, 15].

Extrapulmonary TB is common in HIV-infected patients and can be seen in up to 60 % of HIV-infected patients with TB [16, 17]. Patients with lower CD4 cell counts have the higher risk of extrapulmonary TB and mycobacteremia [18]. The frequent forms of extrapulmonary disease are lymphadenitis, disseminated or bloodstream infection, and TB pleuritis [17–19]. TB meningitis is the most severe form of TB. High mortality rate is observed in spite of ART [20]. Other extrapulmonary sites of HIV-associated TB include bone and joint, skin and soft tissue, pericardium, liver, spleen, kidney, gastrointestinal, and genitourinary tract [21].

### Diagnosis of TB in HIV-infected patients

A definitive diagnosis of TB is confirmed by culturing *M. tuberculosis* organisms from a specimen obtained from the patient. For pulmonary TB, sputum-smear for Ziehl-Neelsen staining is fast, inexpensive, and a highly specific method. However, the sensitivity of direct microscopy in searching for acid-fast bacilli is variable from 31 to 80 % according to a previous review [22]. Studies from Africa found that patients with concurrent HIV and TB infection have a higher frequency of smear-negative TB than those of HIV-uninfected patients [23]. Concentrating the specimen provided an additional positive yield of 36 % for the sputum-negative patients in a high HIV-prevalent area [24]. Currently, sputum microscopy and culture in liquid medium with subsequent drug-susceptibility testing are recommended as standard method for diagnosing active TB [25]. Nevertheless, the use of solid culture medium may be more cost-effective in resource-limited countries. In systematic review, the mean time to detection with Löwenstein-Jensen cultures was 24 days, whereas automated mycobacterial liquid culture systems had a mean time to detection of 15 days [26]. Drug resistant TB substantially reduces survival in HIV-infected patients with TB [27, 28]. Early detection and optimal treatment of drug resistant TB are crucial. Susceptibility testing of *M. tuberculosis* should be performed wherever this test is available.

Beyond sputum microscopy, new molecular diagnostic tests provide a rapid diagnosis of active TB. Xpert MTB/RIF, the automated real-time nucleic acid amplification assay, is endorsed for the diagnosis of pulmonary TB by World Health Organization [29]. Overall pooled sensitivity is 88 % and a pooled specificity is 99 % when used as an initial diagnostic test replacing smear microscopy. However, the pooled sensitivity of Xpert MTB/RIF decreases to 79 % for HIV-infected patients [29]. Other molecular tests, including MTBDRplus and LightCycler Mycobacterium Detection have demonstrated specificities of more than 97 %, but the sensitivities reduced by 6 % when comparing with Xpert MTB/RIF test in HIV-infected patients with pulmonary TB [30]. A tuberculin skin test and an interferon-gamma release assay are unable to distinguish latent TB from active disease [8]. Usefulness of lipoarabinomannan (LAM) antigen-detection assay for the diagnosis of active TB in HIV-infected patients has recently been found to decrease mortality [31–33]. The implementation of LAM testing is likely to offer the greatest benefit in healthcare facilities where diagnostic resources are scarce and patients present with severe illness, advanced immunosuppression, and an inability to self-expectorate sputum [33].

For extrapulmonary disease, Ziehl-Neelsen stain helps to detect acid-fast bacilli in tissues and smears. However, the conventional smear microscopy has limited diagnostic value with low sensitivity [34, 35]. A recent meta-analysis reported that Xpert MTB/RIF has an overall sensitivity of 83.1 % and a pooled specificity of 98.7 % for the diagnosis of extrapulmonary TB [36]. Xpert MTB/RIF is a sensitive diagnostic test for TB detection in lymph node samples (83.1 %) and for the detection of TB meningitis (80.5 %) while only 46.4 % pooled sensitivity was shown in testing with pleural fluid [36]. In tissue samples other than a lymph node, a pooled estimate of sensitivity and specificity were 81.2 and 98.1 %, respectively [37]. However, direct methods sometimes fail to detect mycobacterium in the clinical specimens. Indirect methods sometimes help with diagnosis of extrapulmonary TB. In endemic TB areas, presence of granulomatous inflammation with or without caseation on histopathology is suggestive for TB [38]. Another indirect test such as adenosine deaminase (ADA) test has considerable evidence to support its use for diagnosis of pleural TB and to a slightly lesser extent for TB meningitis [39].

### Integrated therapy of HIV and TB: general concept

Integrated therapy is crucial for HIV-infected patients with TB. The two diseases, HIV and TB, must be managed simultaneously. TB-related mortality in HIV-infected patients is high during the first few months of TB treatment [1]. Additionally, evidence from randomized clinical trials and systemic review has shown that early initiation of ART improves survival of HIV-infected patients with TB [20, 40–46]. Managing the two diseases more effectively during this critical period is therefore essential to improve the patients’ survival and quality of life. The World Health Organization guidelines have recommended ART initiation during this time period [47–49]. However, ART initiation is often delayed due to various factors such as patient characteristics, overlapping drug toxicities, fear of clinicians or patients, and policies of local HIV and TB programs. Delay in initiating ART is more common among patients referred from TB to HIV separate clinics [50]. Delays in the initiation of ART in HIV-infected patients with TB, particularly those with very low CD4 cell counts, are associated with increased mortality risk.

The successful models of integration of care for both HIV and TB mainly use a single facility such as an integrated HIV/TB care center and a single health care provider delivering care for both diseases [51, 52]. The advantages include providing faster initiation of ART treatment for HIV-infected patients with TB, holistic evaluation of the patients, and practical management when patients encounter adverse drug effects. In addition, this model in resource-limited settings is also more feasible to set up, maintain, and train the healthcare providers. A systematic review has demonstrated that integrated care of HIV and TB may overcome challenges such as losing patients to follow-up during the referral process between TB and HIV clinics, burdening patients with increased travel costs and additional time spent in clinics [53]. Interventions for improving adherence and social support can also better reinforce this approach. Integrated therapy for both HIV and TB by the same team of healthcare providers may provide better clinical outcomes and use limited resource more efficiently.

### Combination therapy of TB in general population

Streptomycin became the first effective treatment of TB in 1946, but the treatment was eventually a failure with frequent emergence of streptomycin resistance. The strategy to overcome the resistance problem with combination therapy of streptomycin, aminosalicylic acid, and isoniazid was reported in the 1950s. However, the treatment period was not less than 1 year [54, 55]. After the discovery of rifampin and pyrazinamide, studies showed that the duration of treatment could be shortened to only 6 months by the inclusion of rifampicin and pyrazinamide in the regimen [56, 57]. Currently, a 2-month initial intensive phase with isoniazid, rifampin, and pyrazinamide, followed by 4 months of a continuation phase with isoniazid and rifampin is considered as the standard treatment of drug-susceptible TB [58–60]. Ethambutol is usually recommended as the fourth drug in the intensive phase to prevent unrecognized resistance to one of the three core drugs [61]. Recent clinical trials in shortening TB treatment duration to 4 months by moxifloxacin- or gatifloxacin-containing regimens demonstrated a higher relapse rate than those of standard regimen [62–64].

The multidrug initial intensive phase is to reduce the bacillary load by killing mycobacteria rapidly and to prevent the emergence of drug resistance. The continuation phase of therapy is given to kill the slowly replicating or non-replicating subpopulation. Isoniazid provides bactericidal activity by initially killing about 95 % of bacilli during the first 2 days of treatment. Its bactericidal role is then replaced by rifampicin and pyrazinamide. Rifampicin and pyrazinamide are also responsible for the sterilizing activity by killing persistent or non-replicating organisms [55, 65]. A combination of at least two fully effective drugs was necessary to prevent the emergence of resistance [54].

Pulmonary and extrapulmonary TB should be treated with the same regimens [58]. Six months of standard therapy is generally considered adequate for most forms of extrapulmonary tuberculosis. However, treatment duration may need to be extended for central nervous system and skeletal tuberculosis. Although the optimal duration of therapy cannot be defined, the 9–12 months duration is recommended for the treatment of TB meningitis because of serious risk of disability and mortality and for the treatment of TB of bone and joints because of the difficulties of assessing treatment response [58–60]. Adjunctive corticosteroids reduce the risk of death or disabling residual neurological deficit in patients with tuberculous meningitis [66], and may be beneficial in those with tuberculous pericarditis [67]. An intensified regimen for TB meningitis that includes both a higher dose of oral rifampicin and the addition of levofloxacin to the standard regimen does not provide benefit on reducing overall mortality [68].

### Treatment of TB in HIV-infected patients

The principles for treatment of active TB disease in HIV-infected patients are the same as those for HIV-uninfected patients. TB treatment is the priority. The regimens should include rifampicin and isoniazid [69, 70]. The 6-month duration with standard regimen among HIV-infected patients showed similar cure and treatment failure rates as in HIV-uninfected patients [71–73]. However, a meta-analysis on treatment of active TB in HIV-infected patients suggested that longer duration of rifamycin therapy (at least 8 months) were associated with a lower risk of TB relapse [74]. Intermittent intensive-phase treatment was associated with increased odds of treatment failure [75]. ART was found to lower the risk of TB relapse [74]. Studies to assess duration of rifamycin-based TB treatment and dosing schedule in patients receiving ART are needed for further evaluation. Currently, the World Health Organization’s guideline for the treatment of TB recommends that HIV-infected patients should, as a minimum, receive the same duration and daily dosing of TB treatment as HIV-uninfected patients. ART should be initiated in all HIV-infected with TB patients, irrespective of CD4 cell count [58].

Rifabutin induces CYP3A activity at a lesser magnitude than rifampicin and it may therefore be a more appropriate option for co-administration with many antiretroviral drugs, including PIs [76]. Of noted, PIs increase the dose of rifabutin. Thus, rifabutin dosage required adjustment to minimize rifabutin-associated toxicity, including neutropenia, uveitis, and liver toxicity [59]. A concern with rifabutin 150 mg thrice weekly is that rifabutin under exposure may result in acquired rifamycin resistance. Some experts recommend rifabutin at a dose of 150 mg daily when co-administered with any ritonavir-boosted PI [60, 61]. A recent randomized pharmacokinetic evaluation of different rifabutin doses in African HIV- infected patients with TB and on lopinavir/ritonavir-based ART has demonstrated that a daily 150 mg dose of rifabutin in combination with lopinavir/ritonavir safely maintained rifabutin plasma concentrations [77]. To date, the replacement of rifampicin by rifabutin for first-line treatment of TB is not supported by the current evidence [78].

### ART in HIV-infected patients with TB

The optimal timing to commence ART in HIV-infected patients with TB is relatively complex. It must balance the risk of morbidity and mortality in very advanced HIV disease with the potential occurrence of additive toxicities, drug–drug interactions, and TB-associated immune reconstitution inflammatory syndrome (IRIS). The current World Health Organization guidelines recommend that ART should be started as soon as possible “within the first 8 weeks” of starting antituberculous treatment and within the first 2 weeks for patients who have CD4 cell counts less than 50 cells/mm^3^ [47–49]. The results from randomized control trials have been published over the past 5 years [40–44]. They have shown the same trend of survival benefit of concurrent ART during TB treatment. In addition, ART initiation within the first 2 weeks of TB treatment is beneficial in patients with advanced HIV disease, i.e., CD4 cell count <50 cells/mm^3^ [40–44], although it is associated with a twofold higher frequency of TB IRIS [45]. TB IRIS is generally manageable and survival benefits of early ART in this group outweigh the risk of TB IRIS.

In patients with CD4 cell counts higher than 50 cells/mm^3^, evidence is insufficient to support or refute a survival benefit conferred by early ART initiation [45]. Recently, a randomized control study in co-infected HIV and TB patients with high CD4 cell counts showed no difference of mortality between early and deferred ART on the unfavorable composite endpoint of death, tuberculosis treatment failure, and recurrence [46]. Therefore, patients with less immune deficiency, i.e., CD4 cell counts higher than 50 cells/mm^3^, ART may be deferred until completion of intensive phase of TB treatment without compromising survival. In addition, a study in HIV-associated TB meningitis showed that immediate ART was not associated with reduced 9-month mortality, when compared to deferred ART initiation [20]. The adverse events were significantly more frequently found in both overall and during the first 2 months of treatment in immediate versus deferred ART arm [20]. Nonetheless, treatment guidelines in the resource-rich setting recommends to start ART as soon as possible with close monitoring and prompt management of adverse reactions and central nervous system IRIS [69]. Thus, caution in early ART initiation is warranted in patients with TB meningitis. Table 1 summarizes all randomized control trials of the timing to initiate ART during TB treatment.

**Table 1:** Randomized control trials determining time to initiate ART in HIV-infected patients with TB

Study	TB characteristics	Median CD4 count	Study arms	Mortality difference	Country	ART regimen
(1A) SAPIT 1 Abdool Karim et al. NEJM 2010 [43]	Smear +ve pulmonary TB	150	Integrated vs. sequential	5 vs. 12 deaths/100 PYs 56 % lower in integrated arm	South Africa	ddI, 3TC, EFV
(1B) SAPIT 2 Abdool Karim et al. NEJM 2011 [44]	Smear +ve pulmonary TB	150	4 vs. 8–12 weeks	No differences of AIDS/death Lower in only CD4 <50 8 vs. 26 deaths/100 PYs	South Africa	ddI, 3TC, EFV
(2) CAMELIA Blanc et al. NEJM 2011 [42]	Smear +ve pulmonary TB	25	2 vs. 8 weeks	15 vs. 26 % 38 % lower in 2-week arm	Cambodia	d4T, 3TC, EFV
(3) STRIDE Havlir et al. NEJM 2011 [45]	Confirmed or probable pulmonary TB	77	<2 vs. 8–12 weeks	No differences of mortality Lower in only CD4 <50 15 vs. 27 %	Multi-national	TDF/FTC, EFV
(4) TOROK Torok et al. CID 2011 [49]	TB meningitis	41	<2 vs. 8 weeks	No differences of time to death	Vietnam	AZT, 3TC, EFV
(5) TIME Manosuthi et al. JAIDS 2012 [46]	Confirmed or probable any TB	43	4 vs. 12 weeks	Have a tendency in CD4 <50 10 vs. 14 deaths/100 PYs	Thailand	TDF, 3TC, EFV
(6) TB-HAART Mfinanga et al. Lancet ID 2014 [48]	Culture-confirmed TB	367	≤2 weeks vs. 6 months	No difference between early and late ART on composite endpoint of death, tuberculosis treatment failure, and recurrence	South Africa, Uganda, Zambia, Tanzania	AZT, 3TC, EFV

A rifamycin-containing antituberculous regimen is crucial in the treatment of drug-sensitive TB. The regimens which do not contain rifampicin during the continuation phase are significantly inferior to the standard 6-month regimen in newly diagnosed smear-positive pulmonary TB [79]. Rifapentine, a long-acting rifamycin, is not recommended for TB treatment in HIV-infected patients receiving ART because of significant cytochrome P 450 induction. Both rifampin and rifabutin are less strong hepatic cytochrome P 450 inducers. However, these two drugs upregulate the uridine diphosphate glucuronosyltransferase 1A1 enzymes [80]. Therefore, they are associated with significant drug–drug interactions in different magnitudes with many antiretroviral drugs, including all protease inhibitors (PIs), non-nucleoside reverse transcriptase inhibitors (NNRTIs), and integrase inhibitors. Despite the problem of drug–drug interactions between rifamycins and antiretroviral drugs, simultaneous treatment of both TB and HIV are required. Co-administration of rifampin and PIs is not recommended owing to significantly decreased plasma concentrations of all PIs and poor treatment outcomes [81–83]. Increasing the dosage of ritonavir or other PIs has resulted in unacceptable rates of liver toxicity and intolerability [84, 85]. A small study of double-dose lopinavir–ritonavir in combination with rifampicin-based antituberculosis treatment in South Africa has shown the favourable outcomes, with 80 % of patients achieving TB treatment success. However, 36 % of patients experienced gastrointestinal toxicity and 12 % had elevated liver enzymes [86].

In many resource-limited settings where HIV and TB are epidemic, NNRTI-based ART remains a first-line regimen. Previous studies have shown some effects of rifampicin on the minimal efavirenz concentrations. Some experts recommend increasing the dosage of efavirenz from 600 to 800 mg/day in patients with body weight more than 50 kg to overcome the drug–drug interactions [87]. Subsequent trials have shown that concomitant use of standard-dose efavirenz at 600 mg/day and rifampicin does not compromise the clinical treatment outcomes [88–92]. However, certain genetic polymorphisms of efavirenz metabolism, i.e., single nucleotide polymorphisms of the hepatic cytochrome P450 isoenzyme 2B6 (CYP2B6) gene, have been associated with high inter-patient variations in its plasma concentrations. The *CYP 2B6* 516G>T is associated with high plasma concentrations even among patients concurrently receiving rifampicin [93–96]. Given its proven efficacy and simplicity, the use of a standard dose of efavirenz at 600 mg/day combined with two NRTIs is the recommended regimen in antiretroviral-naïve patients who are receiving rifampicin.

Prior studies demonstrated that rifampicin reduces plasma concentrations of nevirapine by 20–55 % [97–99]. However, virological and immunological responses to nevirapine-based ART when given at a dose of 200 mg twice daily were similar for patients that are and are not receiving rifampin-containing antituberculous treatment [100]. Additionally, a 2-week lead-in period of nevirapine is associated with a higher chance of virological failure owing to subtherapeutic concentration of nevirapine [91]. Non-inferiority of the nevirapine-based ART was not shown in a head-to-head comparison with efavirenz-based ART. With regards to safety, nevirapine at full dose could be a safe, acceptable alternative for patients unable to tolerate efavirenz and other options are not available [101]. A higher dose of nevirapine at 300 mg twice daily to overcome drug–drug interactions is associated with higher rates of liver toxicity [102]. Rifampicin significantly reduces plasma concentrations of rilpivirine and etravirine, thus, these drugs are not recommended to be co-administered with rifampicin [103]. With regards to integrase inhibitor, raltegravir co-administered with rifampicin resulted in lower raltegravir minimum concentration by 61 %. Doubling the dose of raltegravir to 800 mg when co-administered with rifampicin can compensate the effect of rifampicin on raltegravir area under the curve but not overcome the effect on concentration at 12 h after dosing [104].

In the phase II study, high rates of success were achieved at 48 weeks with raltegravir 400 mg twice daily, 800 mg twice daily, or efavirenz 600 mg once daily when used in combination with tenofovir and lamivudine in patients receiving a rifampin-containing TB treatment [105]. However, further larger and long-term studies remain needed to confirm this finding. Given rifampicin lowers the minimum concentration of raltegravir, doubling the dose of raltegravir to 800 mg twice daily should be an appropriate treatment strategy. Studies examining the concomitant use of dolutegravir and rifampicin are ongoing.

The rapid worldwide scale‐up of ART in the resource-limited settings has led to increasing numbers of patients failing first‐line ART. The options for second-line ART after failure with a first-line NNRTI-based regimen generally involve the switch to a PI combined with an alternate NRTI backbone. As aforementioned, the drug–drug interactions between rifampicin and PIs are extensive. Therefore, rifabutin should be an appropriate alternative rifamycin in this setting although clinical data regarding safety and efficacy in the treatment of TB in HIV-infected patients is scanty. However, rifabutin is not available in many countries and it is not produced in fixed dose combination. Thus, an alternative option is using a non-rifamycin anti-TB regimen in HIV-infected patients receiving PIs. Fluoroquinolones such as ofloxacin, levofloxacin, or ciprofloxacin may be used to substitute rifampicin. In addition, a non-rifamycin anti-TB regimen should be given for a period of 9–12 months. The management of HIV-infected patients taking PIs and undergoing treatment for active TB with a non-rifamycin anti-TB regimen should be directed by, or conducted in consultation with, a physician with experience in the care of patients with these two diseases. This care should include close attention to the possibility of TB treatment failure, antiretroviral treatment failure, and overlapping toxicities for all drugs used.

### Overlapping toxicities of antituberculous and antiretroviral drugs

Adverse drug reactions occur more frequently among HIV-infected patients with TB who are receiving concurrent treatment of HIV and TB than HIV-uninfected patients [106, 107]. The common overlapping toxicity profiles include liver toxicity, skin reactions, renal toxicity, and gastrointestinal reactions [108–113]. Table 2 shows the common overlapping adverse events of first-line antituberculous drugs and antiretroviral drugs. These reactions may be complicated and need intensive management in some patients. Early recognition of these adverse events and prompt appropriate management of these problems can lead to successful integrated therapy in HIV-infected patients with TB.

**Table 2:** Common overlapping adverse events of first-line antituberculous drugs and antiretroviral drugs

Adverse events	Antituberculous drugs	Antiretroviral drugs	Clinical characteristics
Skin reaction [108]	Rifamycins, isoniazid, pyrazinamide, ethambutol, streptomycin	Nevirapine, efavirenz, abacavir	Morbilliform rashes, Steven Johnson syndrome and toxic epidermal necrolysis, fixed drug eruption, lichenoid drug eruptions and acute generalized exanthematous pustulosis
Liver toxicity [109–112]	Rifamycins, isoniazid, pyrazinamide	Nevirapine, efavirenz, protease inhibitors	Transaminitis, cholestatic hepatitis
Renal toxicity [113]	Tenofovir	Streptomycin, rifamycins	Tubulo-interstitial nephritis, proximal tubulopathy, acute renal failure

### TB IRIS

**Fig. 1:**
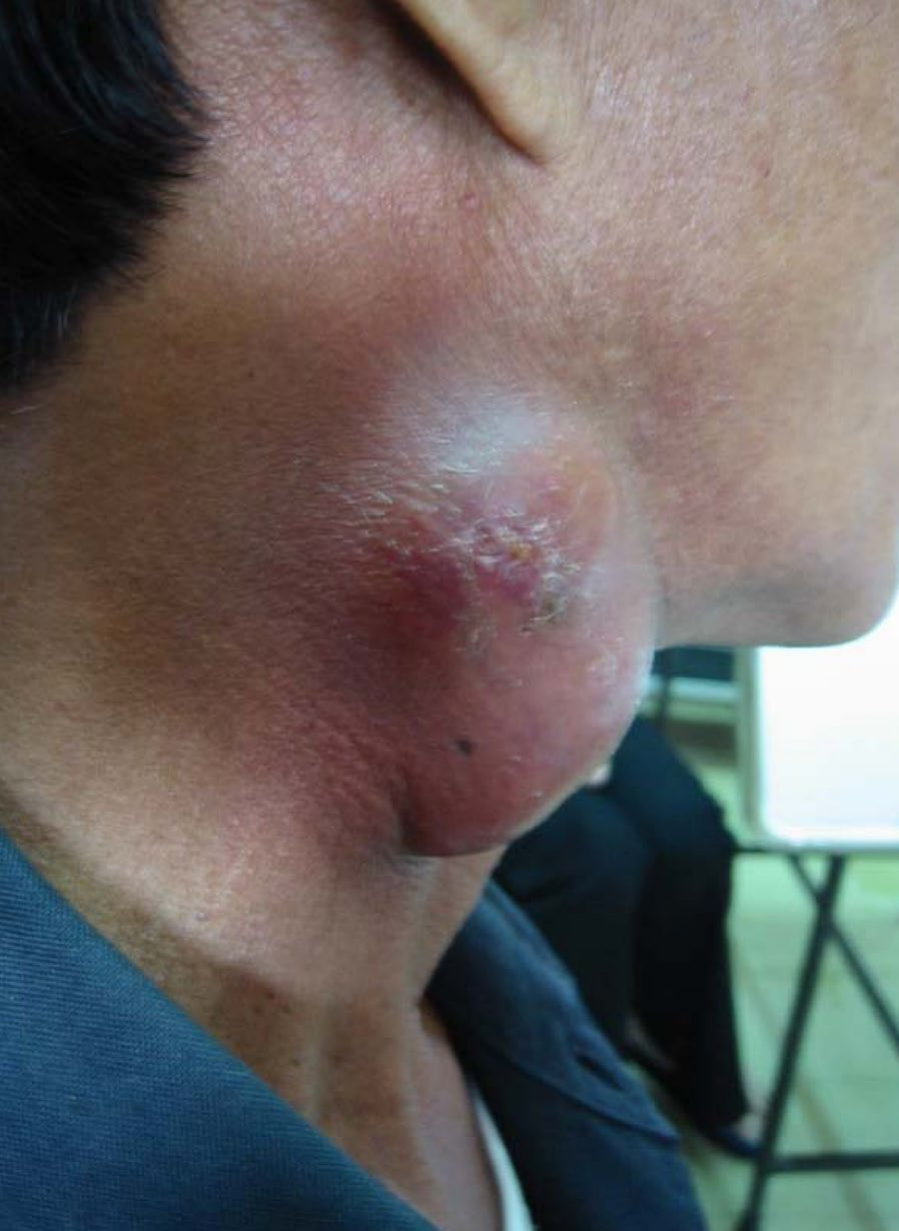
Paradoxical TB IRIS presenting as worsening of lymph node enlargement

TB IRIS may develop among HIV-infected patients who had recently diagnosed active TB or had undiagnosed TB and subsequently received effective ART. TB IRIS or TB-associated immune recovery disease refers to an inflammatory reaction directed to *M. tuberculosis* antigen presenting at the sites of infection. Two patterns of TB IRIS have been described in the literatures, including paradoxical TB IRIS and unmasking TB IRIS. The paradoxical TB-IRIS is defined as the deterioration of existing TB lesions after recent initiation, re-initiation or switch to more effective ART. The patients with paradoxical TB IRIS typically have clinical improvement or clinical stabilization from receiving antituberculous treatment for a short period of time before initiating ART. The epidemiologic data of paradoxical TB-IRIS are variable, ranging from 8 to 42 % [114–118]. However, a meta-analysis showed that the frequency of paradoxical TB IRIS was 15.7 %, with a mortality rate of 3.2 % [119]. The onset of paradoxical TB IRIS typically develops between 1 and 4 weeks after ART commencement and the symptoms lasts for 2–3 months. However, late manifestations of IRIS may present. The common manifestations include high fever, worsening of lymph node enlargement (Fig. 1), and new or worsening of lung infiltration. The neurological TB IRIS may present with cerebral abscess, meningitis, and radiculomyelitis [120, 121]. The identified risk factors of paradoxical TB IRIS include low nadir CD4 cell count at ART initiation [117, 122], extrapulmonary or disseminated TB [116, 123] and a short period between starting TB treatment and ART [118]. The risk factors associated with death in paradoxical TB IRIS included a low nadir CD4 cell count prior to ART initiation and a shorter period between initiating antituberculous treatment and ART [48, 77].

The second pattern, unmasking TB IRIS, occurs after ART initiation in HIV-infected patients with undiagnosed active TB. The subsequent immune restoration provokes the exaggerated symptoms of TB [124]. Thus, the risk factor of unmasking TB IRIS relates to the efficiency of TB screening and diagnosis of subclinical TB prior to ART initiation. A typical clinical presentation is rapid onset and clinical features of TB at the site of infection, such as high fever, pneumonitis, and lymphadenitis [125, 126]. The symptoms may mimic bacterial sepsis.

The consensus case definition of both patterns of TB IRIS is still debated because they do not provide clear consensus on diagnosis [127–129]. The proposed definition of diagnosis in the resource-limited settings has been published previously by the INSHI group [129]. However, patients with undiagnosed multidrug-resistant TB may mimic the symptoms of paradoxical TB IRIS. Thus, antituberculous drug resistance should be excluded in all cases of suspected TB IRIS. In addition, inadequate or inappropriate TB treatment, other concurrent opportunistic infections, and adverse drug reactions need to be excluded. With regard to unmasking TB IRIS, some residual immune deficiency leading to a new TB and deterioration of subclinical TB disease may mimic unmasking TB IRIS [124].

Three major factors involve the pathogenesis of TB-IRIS, including (1) mycobacterial antigen burden and anti-TB treatment, (2) immune system function of the patients, and (3) ART initiation and potency. The role of mycobacterial antigen burden is suggested by the risk of TB IRIS that is greater in the patients with earlier ART initiation during TB treatment when the residual mycobacterial load remains high and in those with disseminated TB [124, 130]. Mycobacterial antigen releasing from the killing bacilli with TB treatment stimulates an exuberant inflammatory response. The host immune system also plays roles in the pathogenesis of TB IRIS. Pre-existing immune deficiency has been shown to predispose to TB IRIS. Many previous studies revealed that the low nadir CD4 cell counts have been related to subsequent IRIS [119, 131, 132]. High plasma HIV viral load has been reported to be associated with TB IRIS [133]. In addition, it is associated with an exuberant production of inflammatory cytokines, such as IFN-gamma [134, 135]. In terms of ART, not only the rate of CD4 cell count recovery in peripheral blood after ART initiation is associated with TB IRIS [115], but the ART may also trigger local immune reconstitution via increased numbers of infiltrating MTB-specific CD4+ T cells at the site of infection [136].

Most cases of paradoxical TB IRIS are self-limited. When mild paradoxical TB IRIS occurs, symptomatic and supportive treatment can be given, including analgesic drugs, anti-pyretic drugs, and anti-emetic drugs. Local drainage of abscess may be required in some cases. Nonsteroidal anti-inflammatory drugs can be used for those cases in which inflammation or fever cause patients discomfort. Moderate to severe TB IRIS may threaten the clinical status of patients, such as neurologic sequelae from TB of central nervous system and decreased pulmonary function of pulmonary TB IRIS. In such cases, anti-inflammatory treatment should be considered. A randomized control trial showed that prednisolone at 1.5 mg/kg/day for 2 weeks followed by 0.75 mg/kg/day for an additional 2 weeks resulted in a reduction of hospitalization and therapeutic procedures [137]. In a case series diagnosed with severe TB IRIS, time to symptom improvement with prednisolone 10–80 mg/day was 3 days [118]. However, adverse effects of corticosteroid treatment should be monitored. ART interruption is not recommended because of increased risk of antiretroviral drug resistance and developing of opportunistic infections. Other potential drugs used to treat TB IRIS are thalidomide, leukotriene receptor antagonists, pentoxifylline, and hydroxychloroquine [138].

## Conclusions

Managing HIV and TB simultaneously is essential to improve patients’ survival and quality of life. Integration of care for both HIV and TB using a single facility and a single health care provider is a model to deliver integrated therapy for both diseases and manage adverse drug effects efficiently. Based on the evidence from randomized clinical trials and systemic review, various treatment guidelines have recommended early ART initiation. Integrated therapy is crucial for HIV-infected patients with TB. For TB treatment, a 2-month initial intensive phase of isoniazid, rifampin, pyrazinamide, and ethambutol, followed by 4 months of a continuation phase of isoniazid and rifampin is considered as the standard treatment of drug-susceptible TB. Rifabutin induces CYP3A activity at a lesser magnitude than rifampicin and it may be a more appropriate option for co-administration of many antiretroviral drugs, including PIs. However, rifabutin is not available in many resource-limited countries—where HIV and TB are epidemic.

ART should be initiated in all HIV-infected patients with TB, irrespective of CD4 cell count. The optimal timing to commence ART in HIV-infected patients with TB is within the first 8 weeks of starting antituberculous treatment and within the first 2 weeks for patients who have CD4 cell counts less than 50 cells/mm^3^. In resource-limited settings, NNRTI-based ART remains a first-line regimen. A standard dose of efavirenz at 600 mg/day combined with two NRTIs is the recommended regimen in antiretroviral-naïve patients who are receiving rifampicin. A standard dose of nevirapine without lead-in could be a safe and acceptable alternative for patients unable to tolerate efavirenz. In the settings where raltegravir is available and accessible, doubling the dose of raltegravir to 800 mg twice daily combined with two NRTIs is recommended. Adverse reactions to either antituberculous or antiretroviral drugs are common in patients receiving integrated therapy. TB IRIS may develop among HIV-infected patients who had recently been diagnosed with active TB, received TB treatment, and subsequently received effective ART. Early recognition and appropriate management of these problems can lead to successful integrated therapy in HIV-infected patients with TB.
